# Epidemiology of hospitalization for hip replacement: Which implications for orthopaedics and rehabilitation? A 15‐year longitudinal study

**DOI:** 10.1002/jeo2.70298

**Published:** 2025-06-01

**Authors:** Umile Giuseppe Longo, Alessandro Mazzola, Alessandro de Sire, Sergio De Salvatore, Giuseppe Salvatore, Mattia Loppini, Antonio Ammendolia, Marco Invernizzi, Stefano Zaffagnini, Kristian Samuelsson, Rocco Papalia, Pieter D'Hooghe

**Affiliations:** ^1^ Fondazione Policlinico Universitario Campus Bio‐Medico Rome Italy; ^2^ Department of Medicine and Surgery, Research Unit of Orthopaedic and Trauma Surgery Università Campus Bio‐Medico Di Roma Rome Italy; ^3^ Physical Medicine and Rehabilitation Unit, Department of Medical and Surgical Sciences University of Catanzaro “Magna Graecia” Catanzaro Italy; ^4^ Research Center on Musculoskeletal Health, MusculoSkeletalHealth@UMG University of Catanzaro “Magna Graecia” Catanzaro Italy; ^5^ IRCCS Ospedale Pediatrico Bambino Gesù Rome Italy; ^6^ Hip Diseases and Joint Replacement Surgery Unit, Humanitas Clinical and Research Center, IRCCS Rozzano Italy; ^7^ Department of Biomedical Sciences Humanitas University Milan Italy; ^8^ Department of Health Sciences University of Eastern Piedmont “A. Avogadro” Novara Italy; ^9^ Translational Medicine, Dipartimento Attività Integrate Ricerca e Innovazione (DAIRI), Azienda Ospedaliera SS. Antonio e Biagio e Cesare Arrigo Alessandria Italy; ^10^ Clinica Ortopedica e Traumatologica II IRCCS Istituto Ortopedico Rizzoli Bologna Italy; ^11^ Department of Orthopaedics, Institute of Clinical Sciences, The Sahlgrenska Academy University of Gothenburg Gothenburg Sweden; ^12^ Orthopaedic Surgeon and Assistant Chief of Surgery for Research, Department of Orthopaedic Surgery and Sportsmedicine Aspetar Hospital Doha Qatar

**Keywords:** epidemiology, hip replacement, orthopaedics, partial, rehabilitation, total

## Abstract

**Purpose:**

The increasing incidence of hip replacement (HR) is related to the ageing of the population and the increasing longevity of new implants. The primary purpose of this study is to assess the number of HR hospitalizations in Italy, referring to official national hospitalization reports. The secondary aim is to evaluate the characteristics of patients who underwent HR, comparing the future predictions of the prevalence of HR between Italy and other countries.

**Methods:**

The present study considered adult patients (aged 20 years or older) who underwent HP. The data regarding hospitalizations for HR performed between 2001 and 2015 were collected by the National Hospital Discharge reports carried out by the Italian Ministry of Health. These reports provided data concerning all hospital admission occurring in Italy, both from public and private institutions.

**Results:**

Overall, 64.5% of HR were performed on female patients, whereas only 35.5% were male. The median length of hospital stay was 12.3 ± 8.1 days. During the 15‐year study period, the main primary diagnoses were osteoarthrosis, localized, primary (46.1%). In 2001, 66,171 hospitalizations for HR were performed, equivalent to 5.4% of all those included in the National Hospital Discharge report (1,228,025); in 2015, this figure reached 95,200 units 7.8% of the total. There has been an annual increase of HR of 2.63%. From 2001 to 2015, the incidence of operations increased from 144.1 to 192.3 per 100,000 residents with at least 20 years of age.

**Conclusions:**

This longitudinal study confirms the progressive incidence in HR hospitalizations and the burden for the healthcare system in orthopaedics and rehabilitation fields.

**Level of Evidence:**

Level II

**Abbreviations**
CI: confidence interval
EPRD: German Arthroplasty Registry
FAI: femoroacetabular impingement
HR: hip replacement
ICD‐9‐CM: International Classification of Diseases, Ninth Revision, Clinical Modification
ISTAT: National Institute for Statistics
RA: rheumatoid arthritis
rTHA: total hip arthroplasty revision
SDO: National Hospital Discharge reports
SPSS: statistical package for social sciences

## INTRODUCTION

Hip replacement (HR) is a highly successful surgical procedure with excellent reported long‐term outcomes [26]. HR is nowadays considered the most effective technique to ensure pain reduction and functional improvement in patients suffering from persistent hip pain and joint stiffness resistant to nonoperative treatments, primarily due to an advanced stage of osteoarthritis (OA) [26].

The noticeable increasing incidence of HR is strongly related to the progressive ageing of the population, which is leading to an increased number of comorbidities and advanced OA, that should be adequately and quickly diagnosed [3]. In this scenario, it should be considered the role of conservative treatments (e.g., hyaluronic acids infiltrations) and rehabilitation before and after surgery for hip OA [4, 5, 15].

Furthermore, the necessity to grant optimal treatments to the patients, which could allow them an efficient functional recovery, is another reason for the significant incidence of HR. Moreover, the rise in HR incidence in different countries is also due to the increasing longevity of new implants, health coverage and health reimbursement policies, new implant material or design (such as those preserving the bone stock), and cutting‐edge technologies [1, 16, 17, 25]. Accordingly, in a recent study by Girardot et al. [7] radiographic results, postoperative modified Harris Hip Score, and postoperative complication rates did not significantly differ between conventional and shorter stems.

Although technological progress allowed the development of reliable and long‐lasting prosthetic equipment, a significant impulse to assess the efficiency of hip prosthetic replacement was made by the ‘National Joint Registries’ [20]. In this scenario, the United Kingdom, Australia, Canada, New Zealand and countries of Northern Europe (Sweden, Norway and Denmark) put their national data at disposal [19]. To date, the diffusion of such national registers is consistently increasing. Despite the variability between individual registers, each database contains patient‐ and surgery‐related information. These data have been mainly used to investigate adverse postoperative outcomes, with particular attention for those leading to revision surgery [30].

Epidemiological data from different national registries could help provide information regarding HR performance, durability and results. Therefore, sharing national statistics and correlating those to other countries could be helpful to compare outcomes and costs internationally. Lastly, National registers allow clinicians to forecast the need for HR to plan economic and health policies [14] in countries with a progressively ageing population [28].

The primary purpose of this study was to assess the number of HR performed in Italy from 2001 to 2015, referring to official national hospitalization reports. The secondary aim was to evaluate the possible variation of patients stratified by age and sex to assess which kind of patient is most inclined to undergo HR. Furthermore, a comparison concerning the actual trend and the future predictions of the prevalence of HR between Italy and other countries has been performed in order to understand the potential implications for Orthopaedics and Rehabilitation fields.

## MATERIALS AND METHODS

### Participants

The present study considered adult patients (aged 20 years or older) who have undergone HR. Furthermore, to investigate the implications associated with age, the patients were divided into 17 different age groups. Each group includes patients born within four years (e.g., 20–24, 25–29, etc.).

### Study design

The data regarding hospitalizations for HR performed between 2001 and 2015 were collected by the National Hospital Discharge reports (SDO) carried out by the Italian Ministry of Health. These reports provided data concerning all hospital admission occurring in Italy, both from public and private institutions. In Italy, the regional authorities are responsible for organizing and supervising healthcare services delivered through local structures (public or private). Data on the healthcare services are collected by hospitals and periodically sent to the Ministry of Health [27]. The register anonymously reports data about sex, age, average days of hospitalization (number of days between a patient's hospitalization date and discharge date), diagnosis and procedure for each patient. Population data were obtained from the National Institute for Statistics (ISTAT) for each year. HR was defined by the following International Classification of Diseases, Ninth Revision, Clinical Modification (ICD‐9‐CM) procedure codes: 81.51 (total HR) and 81.52 (partial HR). Major diagnosis codes were 715.15 (osteoarthrosis, localized, primary), 715.25 (osteoarthrosis, localized, secondary), 820.01 (closed fracture of epiphysis) and 820.02 (closed fracture of mid cervical section). Inclusion criteria: all patients included in the present study underwent a HR procedure. Exclusion criteria: exclusion was applied when a diagnosis code associated with that of HR was atypical and did not apply to the two codes 81.51 (total HR) and 81.52 (partial HR) according to the ICD‐9‐CM.

### Statistical analysis

Descriptive statistical analysis by means of R program (a software environment for statistical computing and graphics) was used to estimate the yearly number of HR, the percentage of males and females, the average age, days of hospitalization, primary diagnoses and primary procedures performed in Italy.

Frequencies and percentages were calculated for categorical variables, means and standard deviations for continuous variables. The annual incidence rates were calculated dividing the number of annual cases by the annual adult population size (people at least 20 years old) obtained from ISTAT. A statutory electronic national population The Statistical Package for Social Sciences (SPSS) version 26 (Armonk, NY: IBM Corp) was used for this data analysis. Tables and graphs were performed using Microsoft Excel (2019) software.

## RESULTS

### Demographics

**Figure 1:**
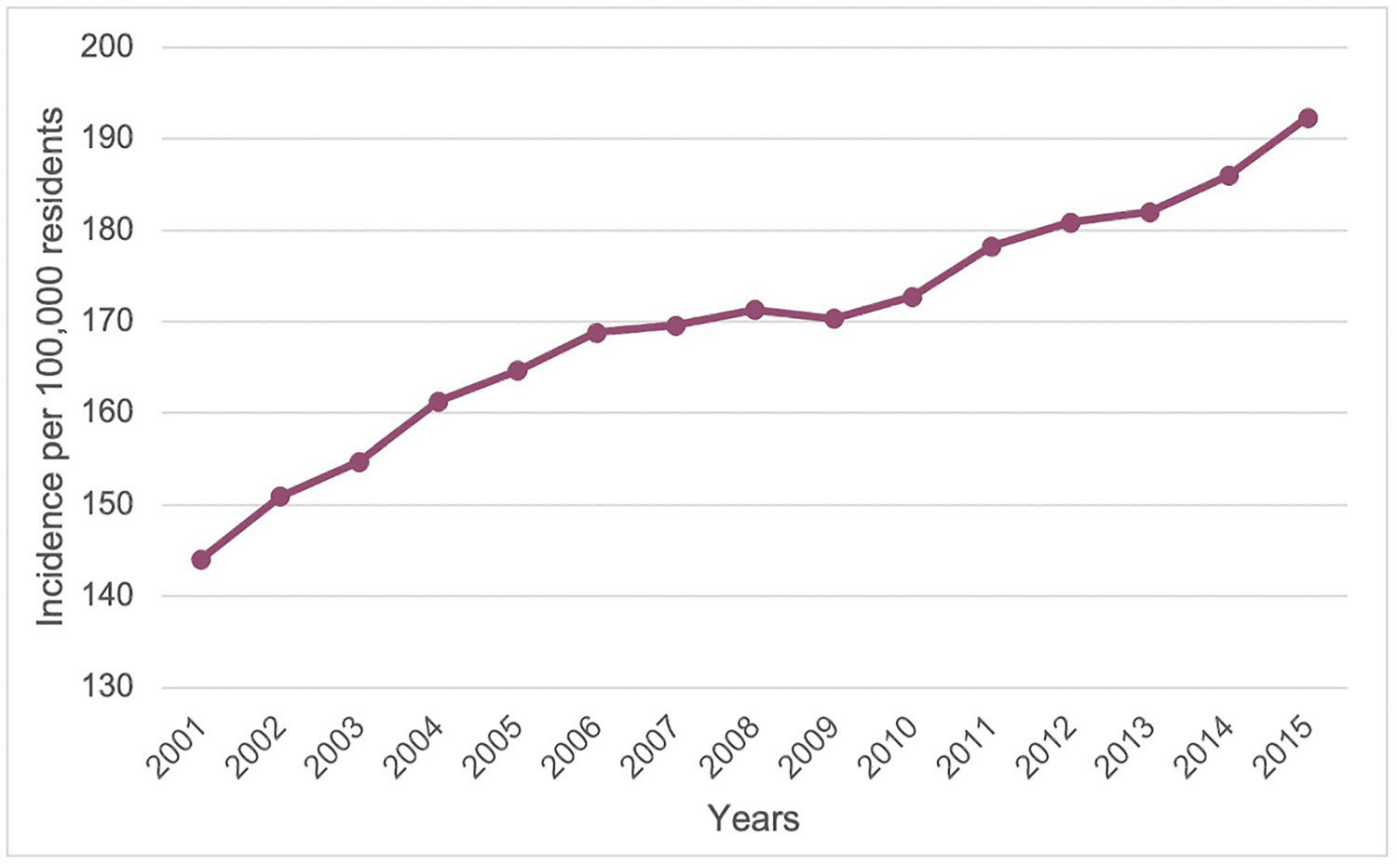
Incidence of hip prosthesis per 100,000 residents.

According to the data collected, there has been a significant increase in HR hospitalizations since 2001. In 2001, 66,171 hospitalizations for HR were performed, equivalent to 5.4% of all those included in the National Hospital Discharge report (1,228,025); in 2015, this figure reached 95,200 units 7.8% of the total. There has been an annual increase of HR of 2.63%. From 2001 to 2015, the incidence of operations increased from 144.1 to 192.3 per 100,000 residents with at least 20 years of age (Figure 1).

**Figure 2:**
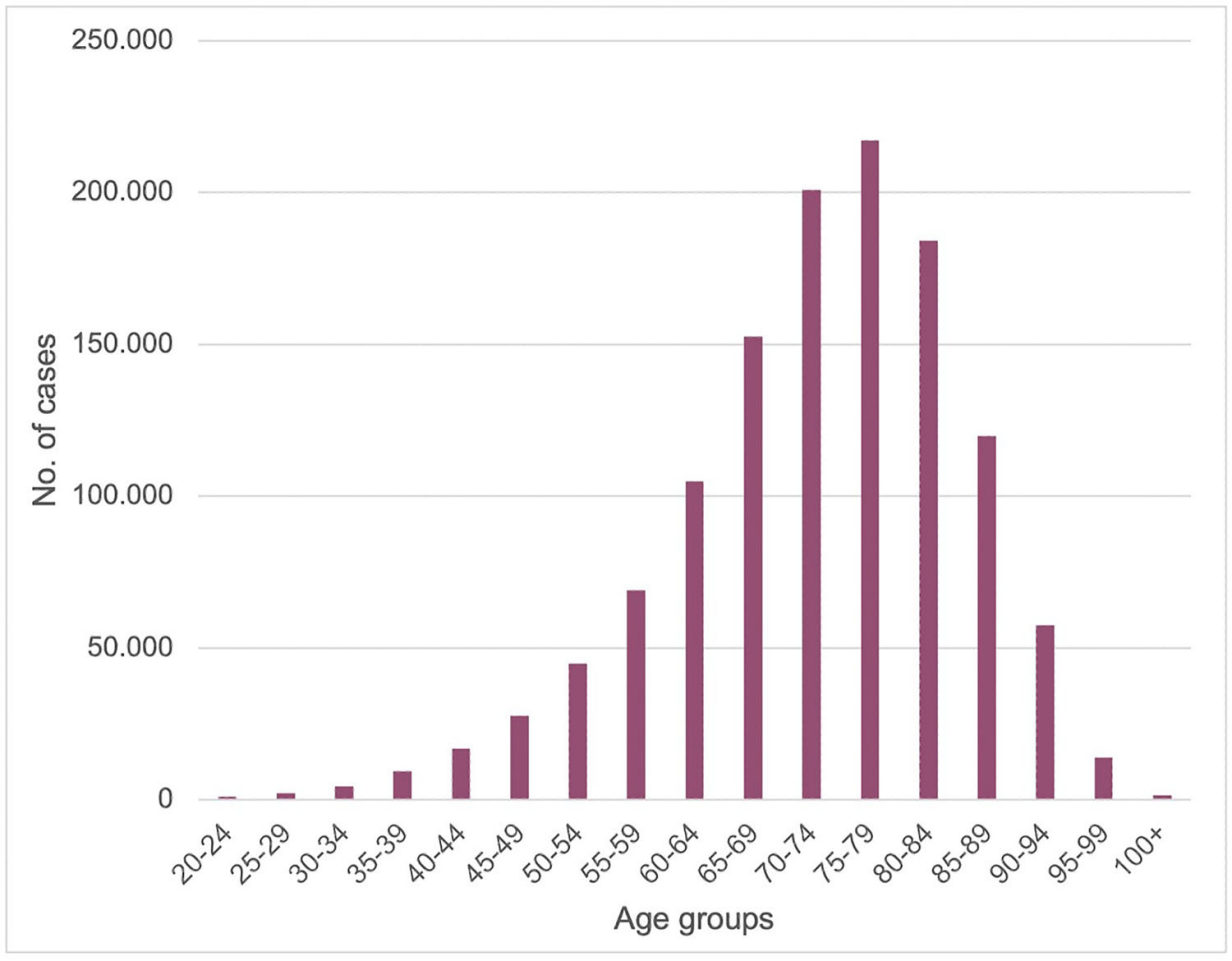
Number of hip prosthesis by age group.

Evaluating the prevalence using the various age groups, the most representative are 70–74, 75–79 and 80–84. Respectively, the patients' percentage was 16.4%, 17.7%, and 15% (Figure 2).

**Figure 3:**
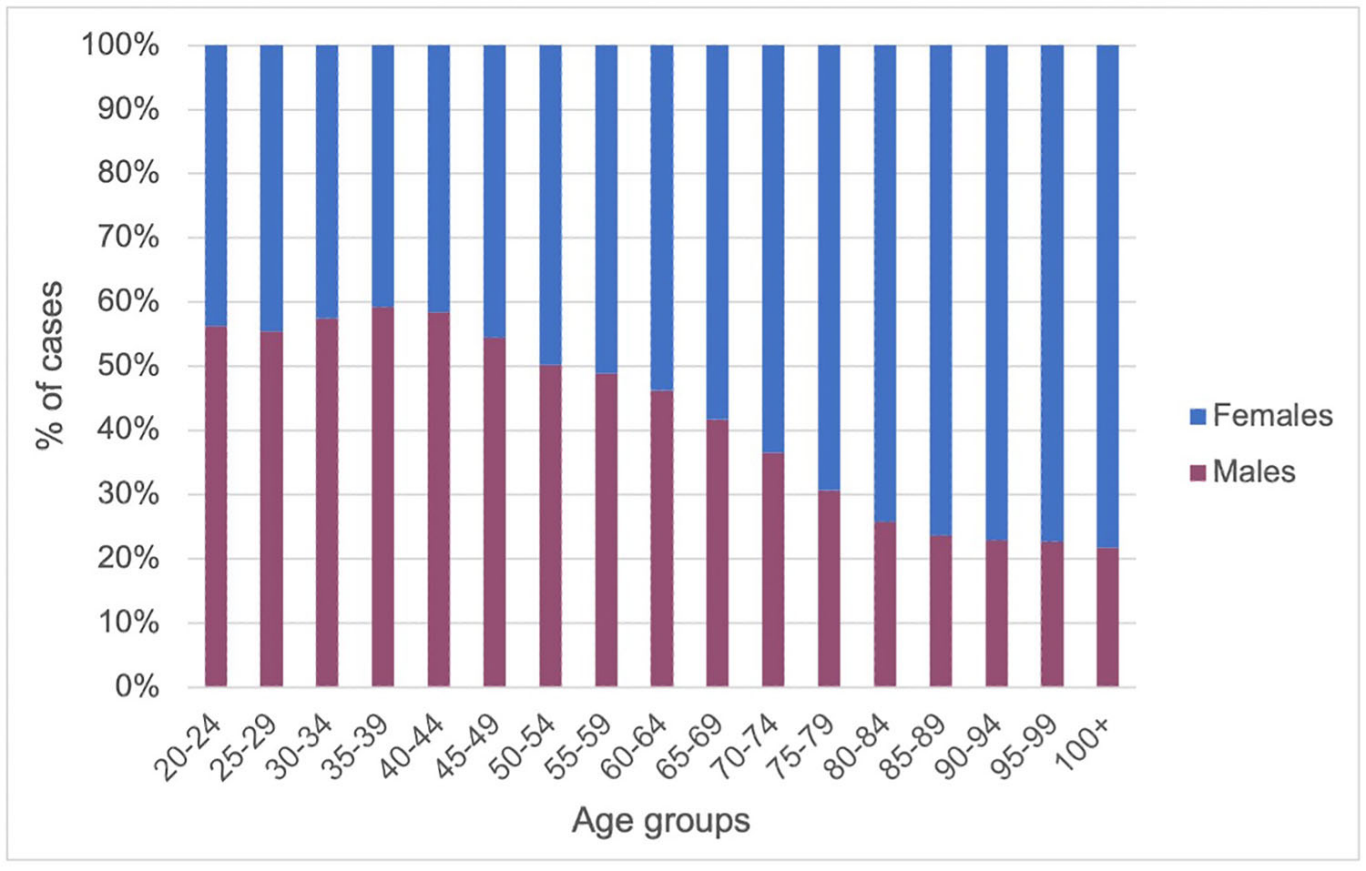
Number of hip prosthesis by age groups and gender.

Of the 1,228,025 patients considered, 64.5% of HR were performed on female patients, whereas only 35.5% were male. In the age groups that include patients aged from 20 to 54, the study showed a higher incidence of a prosthesis in the male patients; on the other hand, in the age groups that include patients over 55 years of age, the incidence was higher in the female patients (Figure 3).

From 2001 to 2015, the average age of patients was 72.5 ± 12.4. The females had an average age higher than males (74.4 ± 11.6 and 69.2 ± 12.9; respectively). The average age of the patients in 2001 was 72.4 ± 12, while the average age of patients in 2015 was 72.7 ± 12.6.

### Length of the hospitalization

**Figure 4:**
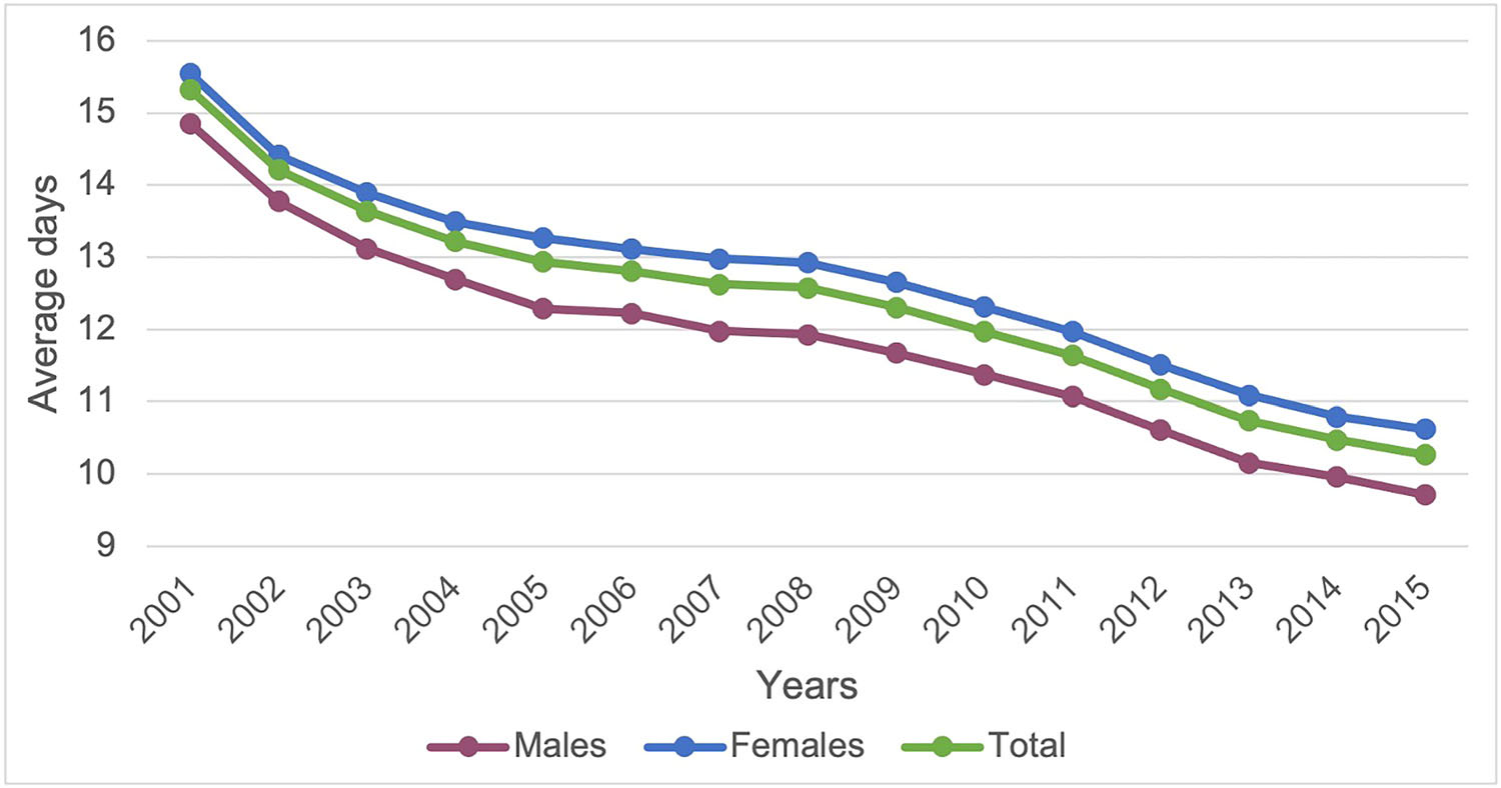
Average days of hospitalization by years.

The median length of hospital stay was 12.3 ± 8.1 days (minimum of 0 days and maximum of 973 days). The trend of the average number of days of hospitalization decreased, from 15.3 ± 9.7 in 2001 to 10.3 ± 69 in 2015 (Figure 4).

Females showed more days of hospital stay than males (12.6 ± 8.1 for women and 11.6 ± 7.9 for men). The oldest age classes (from 70–74 to 100+) showed a higher average of days of hospitalization.

### Most frequent diagnoses

**Figure 5:**
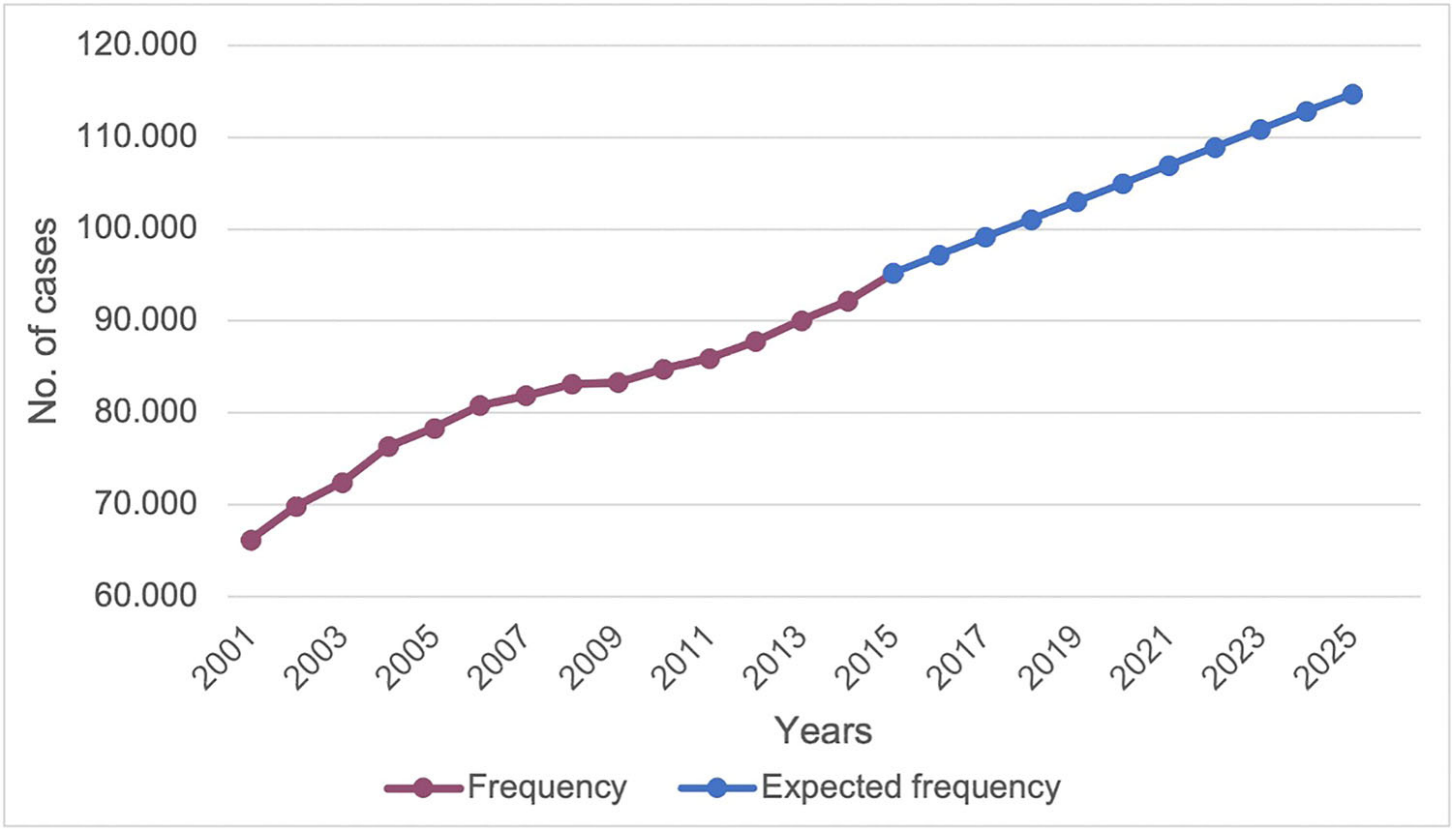
Expected prevalence of hip prosthesis from 2015 to 2025.

During the 15‐year study period, the main primary diagnoses were osteoarthrosis, localized, primary (46.1%, ICD‐9‐CM code: 715.15), closed fracture of epiphysis (12.9%, ICD‐9‐CM code: 820.01), closed fracture of mid cervical section (9.2%, ICD‐9‐CM code: 820.02) and osteoarthrosis, localized, secondary (6.8%, ICD‐9‐CM code: 715.25) (Figure 5).

## DISCUSSION

The present study showed a progressive rising in HR in Italy between 2001 and 2015, with a rate of 1913 prostheses per year, confirming that the socioeconomic burden of HR hospitalization is growing. Therefore, it is essential to predict the future number of HR to assist stakeholders in planning strategies and investments, especially in western countries (where the largest proportion of citizens are elderly).

Adult hip disorders include a broad spectrum of diseases leading to joint deformity and OA. Moreover, HR is the treatment of choice for many types of femoral neck fractures. Two of the main goals of HR are pain relief and function restoration. The leading diagnosis treated with a HR in Italy over the study period was primary hip OA (46.1% of patients). Typical symptoms include a reduction in range of motion (ROM), a shortening of the afflicted limb, and pain and limping that is restricted to the hip, groin, thigh or knee. These statistics highlight the burden of OA, one of the most prevalent chronic illnesses in modern society, which is expected to rise in frequency and incidence as life expectancies rise [23]. The data reported in the present study underlined a significant number of HR in patients aged from 75 and 79. Moreover, it was reported that there was a notable progressive increase in the mean age of patients who underwent HR despite being relatively stable at 73 years between 2010 and 2015. Regarding the variation between gender, a prominent number of female patients who undertook surgery between 2001 and 2015 compared to male patients was reported. Female patients represented 64.5% of total patients, whereas men turned out to be only 35,5%. This evidence also explained the different incidence of HR in male and female patients stratified by age. Some prevalent causes of secondary hip involvement that primarily affect women may partially justify the female predominance in HR, highlighted by the results. At least twice as many women as men suffer from rheumatoid arthritis (RA) [29]. Moreover, young adult women are more likely to experience ‘pincer type’ femoroacetabular impingement (FAI) [6], and female gender is a documented risk factor for hip developing dysplasia [10, 33]. Given their higher life expectancy and superior overall health, women may be more likely than men to have HR in the elderly, as shown by the results of the present study. The hospital length of stay decreased over the 14‐year study period. Under intense financial pressure, the hospitals have reduced hospital length of stay as much as possible, improving ‘fast track’ protocols [32]. By combining evidence‐based clinical and therapeutic methods with organizational optimization, fast‐track HR seeks to give patients the best care possible in a shorter amount of time from admission to discharge [9]. Reducing morbidity and mortality, accelerating the attainment of early postoperative functional milestones, reducing length of stay, and increasing patient satisfaction are the objectives [9]. This result is also due to the significant improvement of prosthetic equipment and surgical techniques over time (e.g., improved anesthesiologic and surgical procedures) [18]. Older patients need, on average, longer hospital stays for the worst general clinical problems.

Reporting national data is essential not only to analyse the epidemiology of HR hospitalization in Italy but also to compare Italian hip arthroplasty trends to other countries. The incidence of HR is not fully understood, especially in Europe. Accordingly, it is hard to make an accurate comparison, considering that national registries' characteristics are very different between countries. A recent study reported an increasing incidence trend of one stage bilateral THA in Italy: [18] demographic features of patients undergoing bilateral one stage HR showed an average age under 60 years and the primary role of hip OA as a leading cause of bilateral hip joint replacement. Interestingly, they reported that only 48% of patients undergoing bilateral one‐stage HR were females. Also in that case, the trend of the mean days of hospital stay decreased over the study period [18]. In 2014, the German Arthroplasty Registry (EPRD) reported 27,595 implanted prostheses. 40% of these operations were performed on male patients, 60% on female patients [11, 20]. Only four years later, the register shows data sets for 150,284 prostheses [11, 20]. This increase can be considered a direct effect of the adhesion of a greater number of hospitals to the project or a real higher incidence of hip prosthetics. There is a clear correlation between patient age and sex distribution in German registries: the older the patient, the lower the proportion of male patients. Most of the operated patients fall within the age group between 75 and 84 years and women are much more represented. This data correlates well with what has been observed in Italy, considering how Germany and Italy have a relatively similar average age (44.4 for Germans and 44.9 for Italians).

Similar results are reported by the International Society of Arthroplasty Registers, the Australian Orthopaedic Association National Joint Replacement Registry or the Swedish national hip arthroplasty register [11, 20].

According to data collected, in Germany, which seems to be more similar to Italy from a demographic point of view, the number of primary HR performed in 2040 was estimated to grow by 27% to 288 × 103 from 2010. According to Pilz et al. [24], the estimated incidence rate was projected to rise from 283 in 2016 to 360 (95% confidence interval [CI]: 312–414) in 2040 per 100,000 German residents. Based on the Nationwide Inpatient Sample data from 1990 to 2004, the demand for primary HR in the United States of America was estimated to grow by 174%, from 208, in 2005 to 572,000 by 2030 [13]. Similar results are available for the United Kingdom [2] and the Nordic countries [8, 21, 22]. In Sweden, according to Nemes et al. [21], a 25% increase in total hip arthroplasty interventions from 2013 to 2030 is expected. Despite these projections, Jensen et al. [12] observed a substantial decline in the rates of hip fractures in Denmark. The authors hypothesized that this dropping trend could be linked to smoking cessation, fall prevention techniques, and better bone health concepts spreading over the country as a result of antiosteoporosis medication (such as bisphosphonates), which entered Danish clinical practice in the mid‐1990s [31]. The lack of compliance with these principles in other countries might partly justify the opposite trend in fracture HR, however no data can confirm this claim.

The reasons for the increase of this surgical procedure worldwide, besides ageing, are connected to the increasing prevalence of OA and the increasing longevity of HR, particularly in young patients.

However, the increased number of HR is directly linked to an expected increase in total hip arthroplasty revision (rTHA) surgery. Culliford et al.^2^ reported that an increase of 32% is expected in the annual rTHA in England. Future research could offer helpful direction for further study, such as studies on revision surgery trends, long‐term patient outcomes, or comparisons with more recent implant technologies.

The present study has several limitations, considering that the ICD‐9‐CM was adopted for all procedures reported. It is a well‐recognized international classification system for diseases, injuries, surgeries, and diagnostic and therapeutic procedures. We adopted the Italian version, and it is mainly used to code the hospital discharge records (SDO).

It is based on administrative records from multiple hospitals in a variety of regions and these data are prone to error. In order to mitigate potential errors, we have stated our inclusion and exclusion criteria: all patients included in the present study underwent HR procedure. Exclusion was applied when a diagnosis code associated with that of HR was atypical and did not apply to the two codes 81.51 (total HR) and 81.52 (partial HR) according to the ICD‐9‐CM.

Second, there are no outcome scores in this study because hospitalizations in Italy's healthcare system are anonymous, and patients are not given an identifiable ID number. Therefore, patients who underwent more than one HR may have been counted more than once. Third, there can be differences between observers because ICD‐9 classification was performed by surgeons. These are very important limitations of the ICD‐9‐CM system. The operative treatment code 81.51 was considered in the present study. The 81.51 code stands for total HR. However, it is a generic code, used for every total HR regardless of the diagnosis. Therefore, this coding system did not allow us to differentiate between HR for fracture and HR for OA performed during the study period. The generic fashion of the ICD9‐CM codes resulted in the impossibility to determine the initial diagnosis in patients undergoing HR.

The ICD‐9‐CM allows for using multiple codes for the same surgical procedure. This heterogeneity in coding could lead to overestimate or underestimate our results. Additionally, the ICD‐9‐CM coding was performed by surgeons, and it may result in individual inter‐observer variations. Moreover, the present study does not compare data from patients undergoing unilateral and bilateral procedures. Lastly, comparing the findings with other countries was difficult due to the differences in healthcare systems.

## CONCLUSIONS

This longitudinal study confirms the progressive incidence in HR hospitalizations. Moreover, the HR burden is relevant for the healthcare system (mainly for the elderly population) in both Orthopaedics and Rehabilitation fields. Comparing statistics from different Nations could help to obtain more homogenous data regarding the survival rate of the implants and outcomes worldwide. Further prospective studies on this topic in different countries can be conducted to make comparisons also in terms of post‐surgery rehabilitation outcomes.

## AUTHOR CONTRIBUTIONS

**Umile Giuseppe Longo:** Conceptualization; investigation; methodology; writing—original draft; supervision. **Alessandro Mazzola:** Investigation; methodology; writing—original draft; writing—review and editing; supervision. **Alessandro de Sire:** Methodology; writing—original draft; supervision. **Sergio De Salvatore:** Methodology; writing—original draft. **Giuseppe Salvatore:** Investigation; visualization. **Mattia Loppini:** Investigation; visualization. **Antonio Ammendolia:** Writing—review and editing. **Marco Invernizzi:** Writing—review and editing. **Stefano Zaffagnini:** Visualization. **Kristian Samuelsson:** Supervision. **Rocco Papalia:** Conceptualization; investigation; methodology; writing—review and editing. **Pieter D'Hooghe:** Formal analysis; visualization.

## CONFLICT OF INTEREST STATEMENT

Kristian Samuelsson is a member of the Board of Directors of Getinge AB (publ) and medtech advisor to Carl Bennet AB. The other authors declare no conflicts of interest.

## ETHICS STATEMENT

The Institutional Review Board of Campus Bio‐Medico University of Rome ruled that no formal ethics approval was required in this particular case and the need to obtain informed consent was waived based on the retrospective design and anonymization of patient identifiers (Prot. number: 113/20 [OSS] ComEt UCBM). All methods were performed in accordance with the relevant guidelines and regulations. All data were obtained by the Direzione Generale della Programmazione Sanitaria—Banca Dati SDO of the Italian Ministry of Health.

## Data Availability

The datasets used and/or analysed during the current study are not publicly available due on our policy statement of sharing clinical data only on request but are available from the corresponding author on reasonable request. The access to the database is on request. All data were obtained by the Direzione Generale della Programmazione Sanitaria—Banca Dati SDO of the Italian Ministry of Health.
